# An Integration of Network Pharmacology and Experimental Verification to Investigate the Mechanism of Guizhi to Treat Nephrotic Syndrome

**DOI:** 10.3389/fphar.2021.755421

**Published:** 2021-12-02

**Authors:** Dan He, Qiang Li, Guangli Du, Guofeng Meng, Jijia Sun, Shaoli Chen

**Affiliations:** ^1^ School of Basic Medicine, Shanghai University of Traditional Chinese Medicine, Shanghai, China; ^2^ School of Pharmacy, Shanghai University of Traditional Chinese Medicine, Shanghai, China; ^3^ Institute of Interdisciplinary Integrative Medicine Research, Shanghai University of Traditional Chinese Medicine, Shanghai, China

**Keywords:** Guizhi, cinnamaldehyde, Nephrotic syndrome, Network pharmacology, MAPK signaling pathway

## Abstract

**Background:** Guizhi has the pharmacological activity of anti-inflammatory. However, the effect mechanism of Guizhi against nephrotic syndrome (NS) remains unclear. A network pharmacological approach with experimental verification *in vitro* and *in vivo* was performed to investigate the potential mechanisms of Guizhi to treat NS.

**Methods:** Active compounds and potential targets of Guizhi, as well as the related targets of NS were obtained from the public databases. The intersecting targets of Guizhi and NS were obtained through Venny 2.1.0. The key targets and signaling pathways were determined by protein-protein interaction (PPI), genes ontology (GO) and kyoto encyclopedia of genes and genomes (KEGG) analysis. And the overall network was constructed with Cytoscape. Molecular docking verification was carried out by AutoDock Vina. Finally, *in vitro* and *in vivo* experiments were performed to verify the mechanism of Guizhi to treat NS.

**Results:** 63 intersecting targets were obtained, and the top five key targets mainly involed in NF- Kappa B and MAPK signaling pathway. In the overall network, cinnamaldehyde (CA) was the top one active compound with the highest degree value. The molecular docking showed that the top five key targets were of good binding activity with the active components of Guizhi. To *in vitro* experiment, CA, the main active component of Guizhi, inhibited the secretion of IL-1β, IL-6, TNF-α in LPS challenged RAW264.7 cells, and down regulated the protein expression of p-NF-κB p65 and p-p38 MAPK in LPS challenged RAW264.7 cells. *In vitro* experiment showed that, 24 urinary protein and renal function were increased in ADR group. To western blot, CA down regulated the protein expression of p-p38 MAPK in rats of adriamycin-induced nephropathy.

**Conclusion:** CA might be the main active component of Guizhi to treat NS, and the underlying mechanism might mainly be achieved by inhibiting MAPK signaling pathway.

## Introduction

Nephrotic syndrome (NS) is a common glomerular disease involving in a variety of causes and it is the second commonly seen kidney disease succeeded to acute glomerulonephritis. Its characterized clinical findings are massive proteinuria, hypoproteinemia, hyperlipidemia and edema (Agrawal et al., 2018). To western medicine, hormones and immunosuppressive therapy are main strategies resorted to the treatment of NS, such as glucocorticoid, cyclophosphamide, cyclosporine, and tacrolimus etc., and certain efficacy is expected. However, being prone to hormone resistance, side effects and recurrence of drug withdrawal are commonly seen in clinic with the treatment of the two therapies, and NS eventually progresses into chronic terminal renal failure (Lombel et al., 2013). Up to now, the pathogenesis of NS remains unknown. More and more reports reveal that it is mainly related to the inflammatory response (Singbartl et al., 2019). Therefore, it is urgent to explore the pathogenesis of NS and find safe and effective approaches.

Studies have shown that the treatment of kidney disease with traditional Chinese medicine (TCM) protected the renal function and delayed the renal failure (Zhang et al., 2018; Zhang B. et al., 2021). Modern pharmacological studies showed that Guizhi (Cinnamomi Ramulus [Lauraceae; Neolitsea cassia]) had the pharmacological activities of anti-inflammatory, etc. (Xu et al., 2013). Wuling Powder, a prescription containing Guizhi, is often used in the treatment of chronic nephritis. Studies have proved that Guizhi is a crucial drug which has a vital role in Wuling Powder (Zhang et al., 2019). Cinnamaldehyde (CA) is the main active component of Guizhi with the pharmacological activities of anti-inflammatory (Kim et al., 2010), antihypertensive (Yao et al., 2015), vascular endothelial protection (Wang et al., 2015), etc., that exerted a protective action on kidney injury in a variety of manners. Our previous study also found that CA had a protective effect on renal function in rats with adriamycin nephropathy (Zhang et al., 2019). However, up to now, the action mechanism of Guizhi to treat NS has not been fully elucidated with suitable approaches.

As a new idea approach of TCM research, network pharmacology, its multi-component, multi-target, multi-pathway thinking method of overall regulation (Zhou et al., 2020) has been applied to predict the main active components, potential targets and action pathways of TCM (Li and Zhang, 2013), and endowing new scientific connotation to traditional Chinese medicine at a new level (Daminelli et al., 2012; Wan et al., 2019).

**FIGURE 1:**
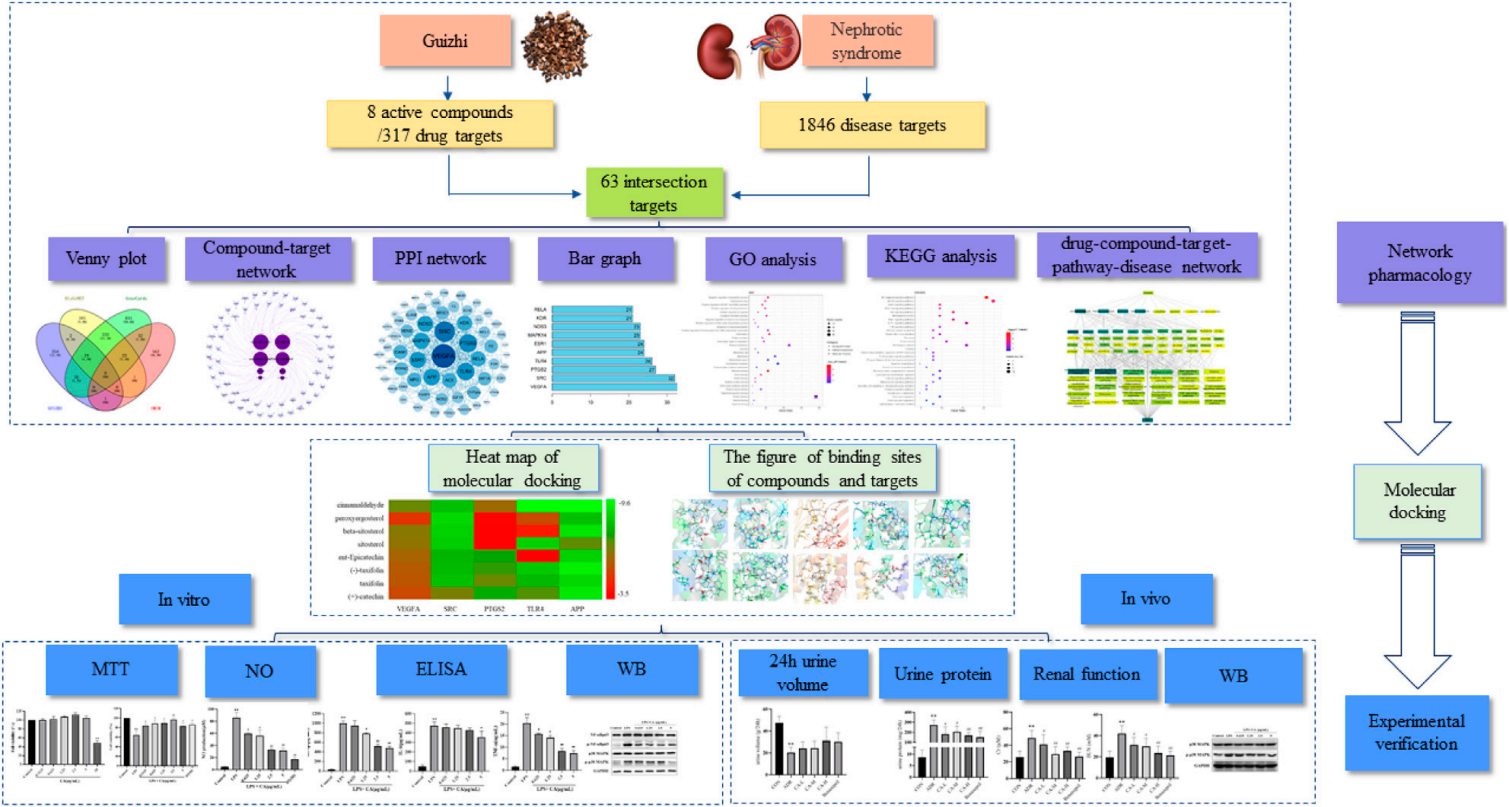
A flow diagram based on a cohesive integration strategy of network pharmacology and molecular docking.

In the present study, the main active components of Guizhi and their possible action mechanism in the treatment of NS were predicted by network pharmacology. In addition, *in vitro* and *in vivo* experiment were also performed to verify the underlying potential mechanism of Guizhi to treat NS. And the flowchart was shown in Figure 1.

## Materials and Methods

### Network Pharmacology Analysis

#### Active Compounds and Drug Targets Screening

The active compounds of Guizhi were searched through Traditional Chinese Medicine Systems Pharmacology Database and Analysis Platform (TCMSP), and the PubChem database was used for confirmation. The PubChem ID, Molecular Formula, Canonical SMILES and other information of each compound were collected, and the main active compounds were screened by oral bioavailability (OB) ≥ 30%, drug-likeness (DL) ≥ 0.18, and cell permeability (Caco-2) ≥ −0.4. HitPick, SEA and Swiss Target Prediction databases were used to make target prediction of the main active components, and the duplicates were deleted after merging the predicted targets of the three databases.

#### Screening of Disease Targets

The targets of NS were predicted using DisGeNET, GeneCards, OMIM databases. After deduplication, Venny 2.1.0 was used to take the intersection with the drug targets, and further identification of intersecting target was carried out through the UniProt database.

#### Construction of Active Compound-Target Network

The active ingredients and the intersecting targets were input into Cytoscape software to construct the active component-target network. Among them, the node size of active ingredient was based on degree value. The larger the node is, the greater the degree value is. Edges were used to indicate the link between the active ingredients and the targets.

#### Construction of PPI Network

The PPI network was constructed by String database and Cytoscape software. The intersecting targets were import into the String database, the parameter of Organism was set as Homo Sapiens, and the protein interaction was obtained. Finally, the PPI network was drawn using the Cytoscape software, and the bar chart of the top 10 intersecting targets was drawn according to the degree values.

#### GO and KEGG Pathway Enrichment

The GO enrichment and KEGG pathway analysis of the intersecting targets were performed using DAVID database. The intersecting targets were imported into the DAVID database, and the threshold was set as *p* < 0.05. After the GO items (biological process (BP), cellular component (CC), molecular function (MF)) and KEGG signaling pathways were filtered, the corresponding bubble diagram of the top 10 BP, CC, MF items and top 30 KEGG signaling pathways was drawn according to the *p* values.

#### Construction of Overall Network

The Guizhi and its active compounds, top 30 KEGG signaling pathways and its targets, as well as NS were used to construct the overall network by Cytoscape.

#### Molecular Docking

The top five intersecting targets, selected from the PPI network, and the main active compounds of Guizhi were selected for the molecular docking through Autodock Vina. The smaller the binding energy (affinity), the more stable the interaction between target and active component is.

### Experimental Verifications *in Vitro*

#### Reagents

LPS and CA were purchased from Sigma-Aldrich, Inc. (St. Louis, United States). DXMS was purchased from Shanghai Yuanye Biotechnology Co., LTD. Griess kit and ELISA kits were purchased from MULTISCIENCES (LIANKE) BIOTECH, CO., LTD. (Hangzhou, China). Antibody: NF-κB p65, p-NF-κB p65, p38 MAPK and p-p38 MAPK were purchased from Cell Signaling Technology, Inc. (Danvers, Massachusetts, United States).

#### Cell Culture

RAW264.7 cells were cultured in DMEM high glucose medium with 10% fetal bovine serum at 37°C, 5% CO_2_ atmosphere. Experiments were carried out when the cells had grown to about 80%.

#### MTT Assay

MTT assay was used to evaluate the cytotoxicity and cell proliferation ability of C on RAW264.7 cells. After inoculated into 96-well plates at a density of 5 × 10^3^ cells /mL overnight, RAW264.7 cells were pretreated with or without CA of different concentrations and DXMS (10 μg/ ml) for 2 h, and then co-incubated with or without LPS (1 μg/ ml) for 24 h. After 20 μL MTT solution was added to each well for 4 h, the supernatant was removed, and 150 μL DMSO was added. The OD value was measured by a microplate analyzer at 570 nm. Each group had five replicates and repeated for three times.

#### Griess Assay of NO

RAW264.7 cells were seeded in 6-well plates at a density of 3 × 10^5^ cells per well. After overnight incubation, cells were pretreated with or without various concentrations of CA and DXMS (10 μg/ ml) for 2 h, and then co-incubated with or without LPS (1 μg/ ml) for 24 h. The levels of NO in cell culture supernatants were measured using Griess kit according to the manufacturer’s instructions. OD value was detected at 450nm, and standard curve was made to obtain the content of the above factor.

#### ELISA Assay

The concentrations of IL-1β, IL-6, and TNF-α in the cell culture medium were detected by ELISA kits. After cell intervention, the supernatant was taken for detection according to the kit instructions, and then detected the OD value and made the standard curve.

#### Western Blot

After intervention for 24 h, total protein was extracted from each group, and the protein concentration was measured by BCA method. 10 μL of sample loading was taken from each group, 10% SDS PAGE gel electrophoresis was performed, PVDF film transfer, milk powder sealed for 2h, TBST film washing, primary antibody (1:1000) was incubated overnight at 4°C, TBST film washing, secondary antibody (1:1000) was incubated for 4h, TBST film washing, exposure.

### Experimental Verifications *in Vivo*

#### Reagents

Adriamycin (ADR) was purchased from Shanghai Wokai Chemical Reagent Co., Ltd (shanghai, China); CA was purchased from Sigma-Aldrich, Inc (St. Louis, United States); Benazepril hydrochloride was purchased from Beijing Novartis Pharmaceutical Co., Ltd (beijing, China); Urea nitrogen kit and creatinine kit were purchased from CHANGCHUN HUILI BIOTECH CO., Ltd (changchun, China); Urinary protein quantitative kit was purchased from Nanjing Jiancheng Bioengineering Institute (nanjing, China); HE staining fluid was purchased from Wuhan Google Biotechnology Co. Ltd (wuhan, China); Primary antibody: p38 MAPK, p-p38 MAPK and GAPDH were purchased from Cell Signaling Technology, Inc. (Danvers, Massachusetts, United States); Secondary antibody was purchased from Beyotime Biotechnology Co., Ltd (shanghai, China).

#### Animals

A total of 36 male SD rats were randomly divided according to body weight, including six rats in the normal group, and the other 30 rats were injected with ADR (3 mg/ kg) via tail vein on day 1 and 8, respectively, to create a model of ADR-NS in SD rats. After successful modeling, they were randomly divided into six groups as follows: control (CON), ADR, CA low-dose + ADR (CA-L), CA middle-dose + ADR (CA-M), CA high-dose + ADR (CA-H) and Benazepril + ADR (Benazepril), with six rats in each group. Each group was given corresponding drugs at 10 mg/ kg dose, and CON and ADR groups were given equal volume of normal saline once a day for 28 days.

#### 24 h Urine Volume and Urine Protein

Urine of rats in each group was collected in the metabolic cage for 24 h, and the specific urine volume was determined. The urine was centrifuged for supernatant, and the urine protein content was detected according to the kit instructions.

#### Renal Function

Blood samples from abdominal aorta were centrifuged and plasma creatinine and urea nitrogen were detected by automatic biochemical analyzer.

#### Western Blot

The kidney was removed and frozen in a −80°C refrigerator. After protein quantification using BCA, the protein expressions of P38 MAPK and P-P38 MAPK were detected by Western blot.

#### Statistical Analysis

GraphPad Prism version 8.0.1was used for descriptive statistical analyses. Data were analyzed by One-way ANOVA, Tukey method, Kruskal-Wallis H rank sum test and the T^2^ test, and *p*<0.05 was considered to be statistically significant.

## Results

### Network Pharmacology Analysis

#### Active Compounds, Drug Targets of Guizhi

A total of 220 compounds of Guizhi were included in TCMSP database, among them, seven main active compounds were preliminarily screened out using ADME parameters, as OB ≥ 30%, DL ≥ 0.18 and Caco-2 ≥ -0.4. cinnamaldehyde (CA), with OB = 31.99%, DL = 0.02, Caco-2 = 1.35, was not in the main active compounds. However, our previous study found that CA might be an important bioactive component of Guizhi (Zhang et al., 2019). Therefore, CA was selected as a candidate active component in this study, as shown in Table 1. Eight major active components of Guizhi were input into Hitpick, SEA and Swiss Target Prediction database, and 7, 89, 261 targets were collected, respectively. After merging the predicted targets from the three databases, the duplicates were deleted, and a total of 317 potential targets were screened out.

**TABLE 1:** The main active compounds of Guizhi.

Mol ID	Molecule name	OB (%)	DL	Caco-2
MOL001736	(-)-taxifolin	60.51	0.27	-0.24
MOL000358	beta-sitosterol	36.91	0.75	1.32
MOL000359	sitosterol	36.91	0.75	1.32
MOL000492	(+)-catechin	54.83	0.24	-0.03
MOL000073	ent-Epicatechin	48.96	0.24	0.02
MOL004576	taxifolin	57.84	0.27	-0.23
MOL011169	peroxyergosterol	44.39	0.82	0.86
MOL000991	cinnamaldehyde	31.99	0.02	1.35

#### NS Related Disease Targets

**FIGURE 2:**
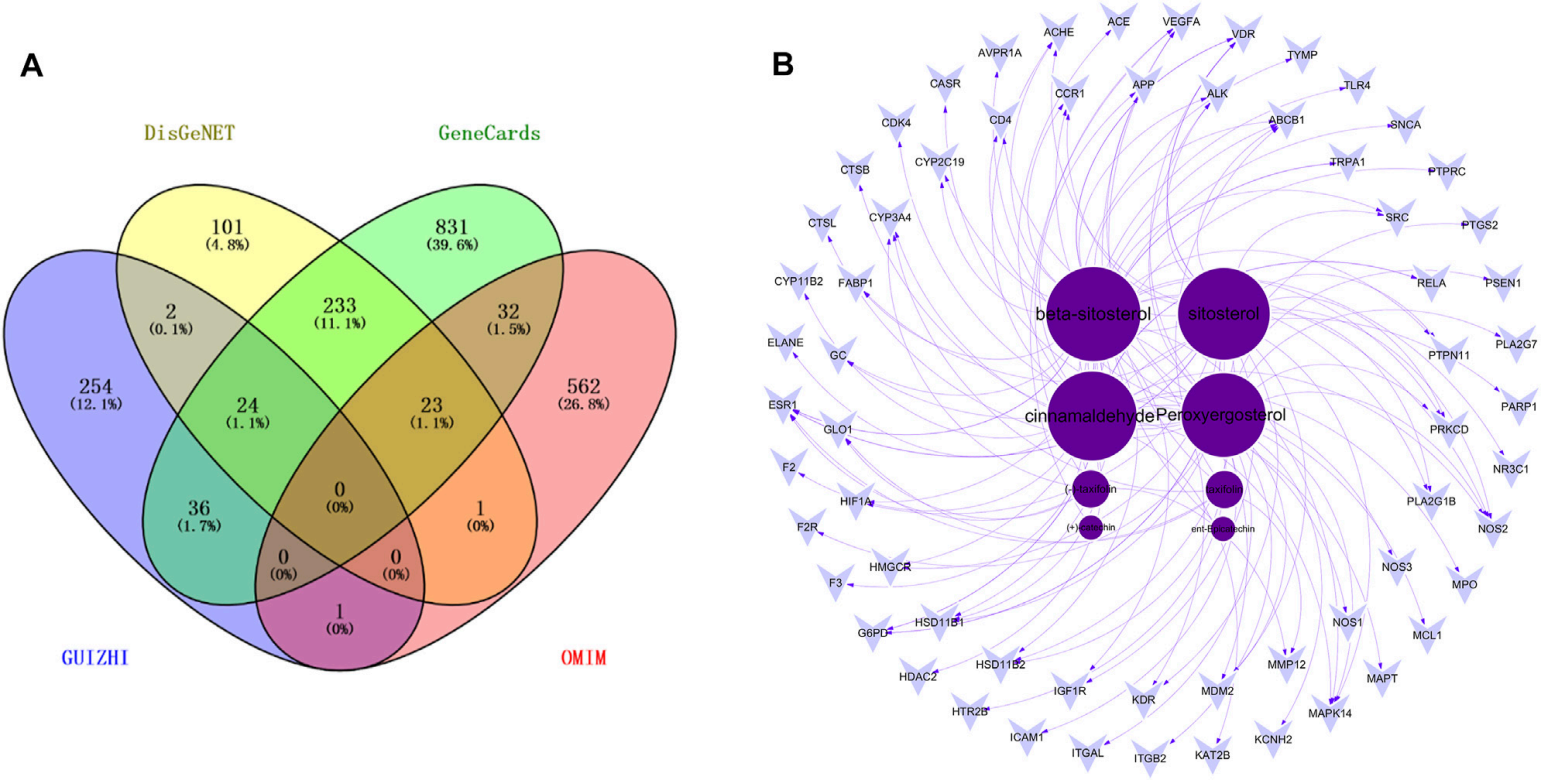
The Venny plot of 63 intersecting targets **(A)** and the active component-target network **(B)**.

With “Nephrotic syndrome” as the key words, 384, 1179 and 619 related disease targets were predicted in DisGeNET, GeneCards, OMIM databases, respectively. The three sets of data were combined and the duplicates were removed. A total of 1846 targets were obtained, and Venny 2.1.0 was used to intersect disease targets with drug targets. Finally, 63 intersecting targets of Guizhi that might act on NS were obtained (Figure 2A). All the 63 intersecting targets were further confirmed by UniProt database, and the results were shown in Table 2.

**TABLE 2:** 63 intersecting targets and UniProt information.

NO	Gene names	Protein names	UniProt ID
1	ABCB1	ATP-dependent translocase ABCB1	P08183
2	ACE	Angiotensin-converting enzyme	P12821
3	ACHE	Acetylcholinesterase	P22303
4	ALK	ALK tyrosine kinase receptor	Q9UM73
5	APP	Amyloid-beta precursor protein	P05067
6	AVPR1A	Vasopressin V1a receptor	P37288
7	CASR	Extracellular calcium-sensing receptor	P41180
8	CCR1	C-C chemokine receptor type 1	P32246
9	CD4	T-cell surface glycoprotein CD4	P01730
10	CDK4	Cyclin-dependent kinase 4	P11802
11	CTSB	Cathepsin B	P07858
12	CTSL	Procathepsin L	P07711
13	CYP11B2	Cytochrome P450 11B2	P19099
14	CYP2C19	Cytochrome P450 2C19	P33261
15	CYP3A4	Cytochrome P450 3A4	P08684
16	ELANE	Neutrophil elastase	P08246
17	ESR1	Estrogen receptor	P03372
18	F2	Coagulation factor II	P00734
19	F2R	Coagulation factor II receptor	P25116
20	F3	Coagulation factor III	P13726
21	FABP1	Fatty acid-binding protein 1	P07148
22	G6PD	Glucose-6-phosphate 1-dehydrogenase	P11413
23	GC	Group-specific component	P02774
24	GLO1	Glyoxalase I	Q04760
25	HDAC2	Histone deacetylase 2	Q92769
26	HIF1A	Hypoxia-inducible factor 1-alpha	Q16665
27	HMGCR	3-hydroxy-3-methylglutaryl-coenzyme A reductase	P04035
28	HSD11B1	Corticosteroid 11-beta-dehydrogenase isozyme 1	P28845
29	HSD11B2	Corticosteroid 11-beta-dehydrogenase isozyme 2	P80365
30	HTR2B	5-hydroxytryptamine receptor 2B	P41595
31	ICAM1	Intercellular adhesion molecule 1	P05362
32	IGF1R	Insulin-like growth factor 1 receptor	P08069
33	ITGAL	Integrin alpha-L	P20701
34	ITGB2	Integrin beta-2	P05107
35	KAT2B	Histone acetyltransferase KAT2B	Q92831
36	KCNH2	Potassium voltage-gated channel subfamily H member 2	Q12809
37	KDR	Kinase insert domain receptor	P35968
38	MAPK14	Mitogen-activated protein kinase 14	Q16539
39	MAPT	Microtubule-associated protein tau	P10636
40	MCL1	Induced myeloid leukemia cell differentiation protein Mcl-1	Q07820
41	MDM2	E3 ubiquitin-protein ligase Mdm2	Q00987
42	MMP12	Matrix metalloproteinase-12	P39900
43	MPO	Myeloperoxidase	P05164
44	NOS1	Peptidyl-cysteine S-nitrosylase NOS1	P29475
45	NOS2	Peptidyl-cysteine S-nitrosylase NOS2	P35228
46	NOS3	NOS type III	P29474
47	NR3C1	Nuclear receptor subfamily 3 group C member 1	P04150
48	PARP1	Poly [ADP-ribose] polymerase 1	P09874
49	PLA2G1B	Phosphatidylcholine 2-acylhydrolase 1B	P04054
50	PLA2G7	Platelet-activating factor acetylhydrolase	Q13093
51	PRKCD	Protein kinase C delta type	Q05655
52	PSEN1	Presenilin-1	P49768
53	PTGS2	Prostaglandin G/H synthase 2	P35354
54	PTPN11	Tyrosine-protein phosphatase non-receptor type 11	Q06124
55	PTPRC	Receptor-type tyrosine-protein phosphatase C	P08575
56	RELA	Transcription factor p65	Q04206
57	SNCA	Alpha-synuclein	P37840
58	SRC	Proto-oncogene tyrosine-protein kinase Src	P12931
59	TLR4	Toll-like receptor 4	O00206
60	TRPA1	Transient receptor potential cation channel subfamily A member 1	O75762
61	TYMP	Thymidine phosphorylase	P19971
62	VDR	Vitamin D3 receptor	P11473
63	VEGFA	Vascular endothelial growth factor A	P15692

#### Analysis of Active Component-Target Network

63 intersecting targets were input into Cytoscape software to construct the active component-target network (Figure 2B). Nodes represented active components or intersecting targets (deep purple circles represented active components, light purple inverted triangles represented intersecting targets), and the degree value was represented by the node size, the larger the node, the greater the degree value. The edges represented the connection between active components and intersecting targets. As can be seen from the figure, different active components corresponded to different targets, which reflected the characteristics of multi-component, multi-target of Guizhi. Among them, the degree values of beta-sitosterol, sitosterol, cinnamaldehyde and peroxyergosterol were 28, 27, 26 and 24, respectively, which indicated that they were the most important active components in the network (Table 3).

**TABLE 3:** Degree value of eight main active components of Guizhi.

Mol ID	Molecule name	Degree
MOL000358	beta-sitosterol	28
MOL000359	sitosterol	27
MOL000991	cinnamaldehyde	26
MOL011169	peroxyergosterol	24
MOL001736	(-)-taxifolin	6
MOL004576	taxifolin	6
MOL000492	(+)-catechin	1
MOL000073	ent-Epicatechin	1

A total of 317 drug targets of Guizhi were screened out in Hitpick, SEA and Swiss Target Prediction database, 384, 1179 and 619 disease targets were predicted in DisGeNET, GeneCards, OMIM databases, respectively. Venny 2.1.0 was used to intersect the drug targets with disease targets. Finally, 63 intersecting targets were obtained. Then, the 63 intersecting targets were input into Cytoscape software to construct the active component-target network. The deep purple circles represented active compounds, light purple inverted triangles represented intersecting targets, and the degree value was represented by the node size. Among them, the degree values of beta-sitosterol, sitosterol, cinnamaldehyde and peroxyergosterol were 28, 27, 26, and 24, respectively, which indicated that they were the most important active components in the network.

#### PPI Network Analysis

**FIGURE 3:**
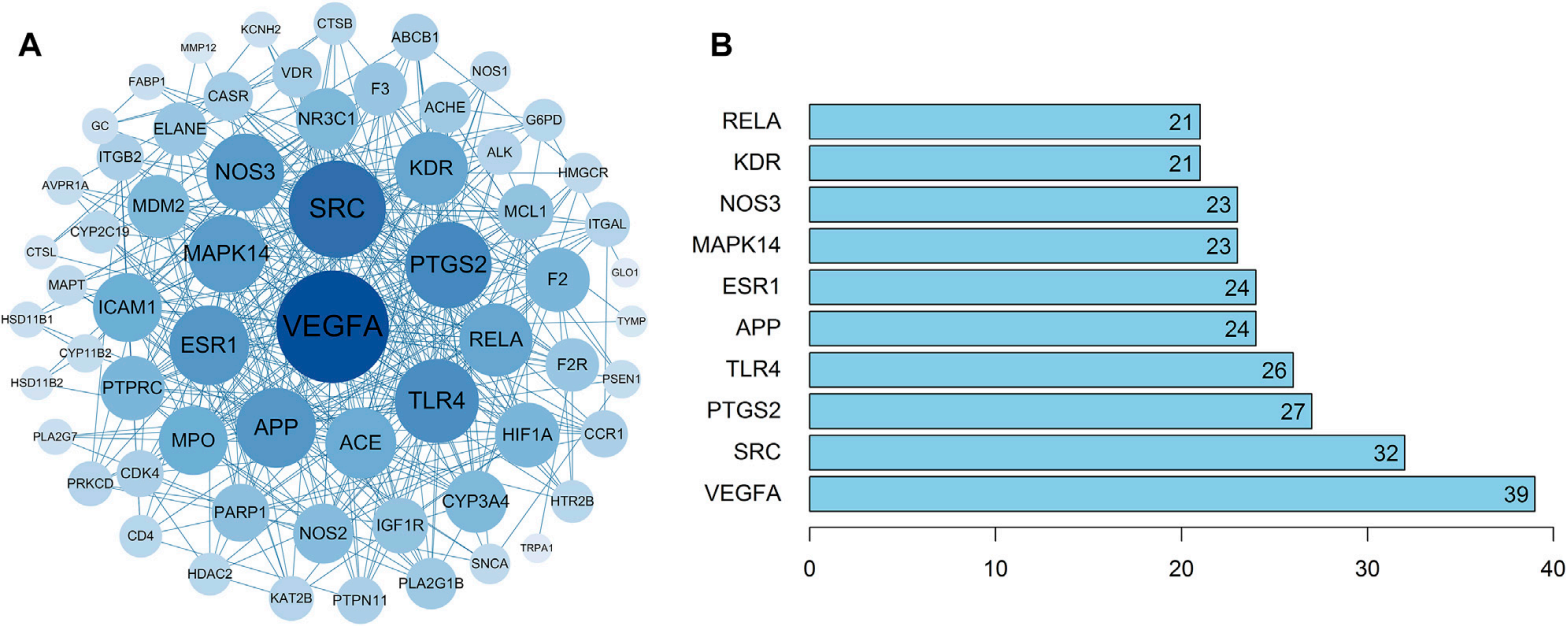
The PPI network of the 63 intersecting targets **(A)** and the top ranked proteins **(B)**. The 63 intersecting targets were imported into Cytoscape to construct the PPI network, the size and color of the nodes reflected the values of degree. Among them, the degree values of VEGFA, SRC, PTGS2, TLR4 and APP were 39, 32, 27, 26, and 24, respectively, which were the key nodes of the network.

The 63 intersecting targets were imported into the String database, and the obtained information was imported into Cytoscape software to construct PPI network (Figure 3A). Nodes represented intersecting targets and edges represented the interactions between targets. The figure involved a total of 63 nodes and 370 edges. The size and color of the nodes reflected the values of degree. A bar chart of the top 10 intersecting targets was drawn according to the degree value (Figure 3B). Among them, the degree values of VEGFA, SRC, PTGS2, TLR4 and APP were 39, 32, 27, 26, and 24, respectively, which were the key nodes of the network, suggesting that Guizhi might play an effective role against NS through them.

#### GO and KEGG Analysis

**FIGURE 4:**
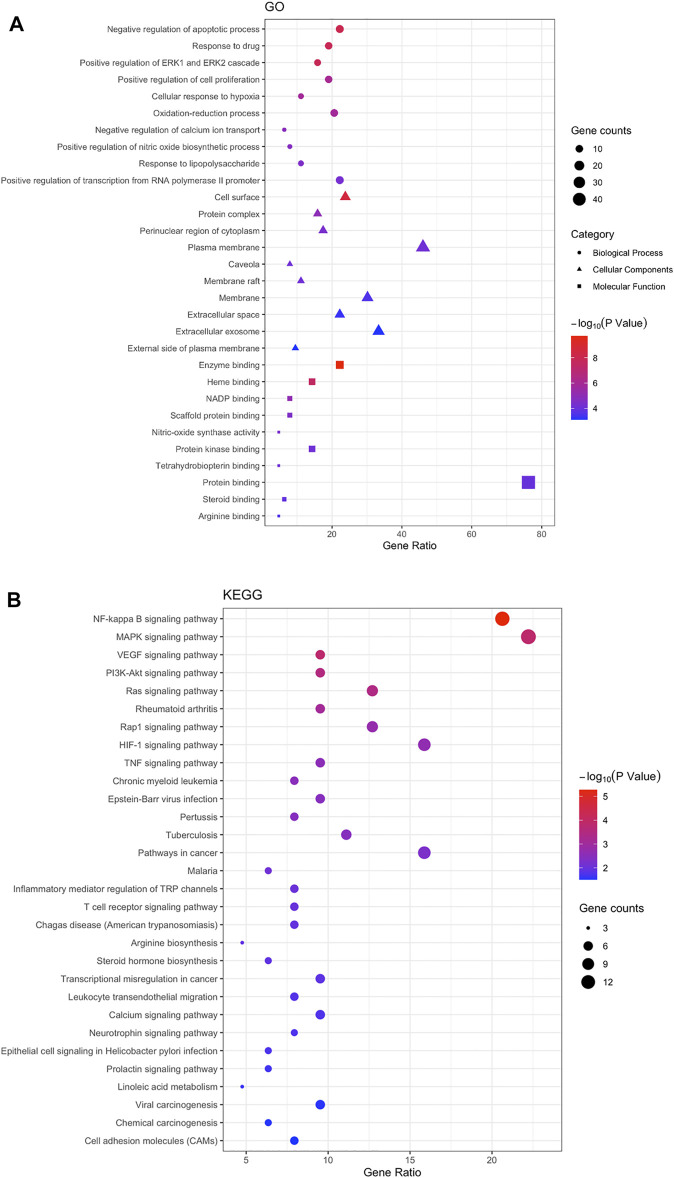
The Go **(A)** and KEGG **(B)** enrichment analysis of the targets. 63 intersecting targets were imported into DAVID to screen GO entries (BP, CC, MF) and KEGG signaling pathways with *p* < 0.05. A total of 150 BP, 31 CC, 46 MF and 35 signaling pathways were predicted, and we found that most of the key intersecting target were involved in the NF-Kappa B and MAPK signaling pathways.

63 intersecting targets were imported into DAVID database to screen GO entries (BP, CC, MF) and KEGG signaling pathways of *p* < 0.05. A total of 150 BP related items were found by GO functional enrichment analysis, which mainly involved negative regulation of apoptotic process, response to drug and other biological processes. There were 31 items related to CC, mainly involving cell surface, protein complex and other cellular components. 46 items related to MF, mainly involved enzyme binding, nitric-oxide synthase activity and other molecular functions (Figure 4A). The results indicated that NS, as a complex disease, involves a variety of biological processes, and Guizhi might play a protective role against NS by regulating these biological processes. KEGG signaling pathway analysis showed that the intersecting targets mapped 35 signaling pathways, mainly involving NF-Kappa B, MAPK and other signaling pathways. We further found that most of the key intersecting target were involved in the NF-Kappa B and MAPK signaling pathways (Figure 4B).

#### Overall Network Analysis

**FIGURE 5:**
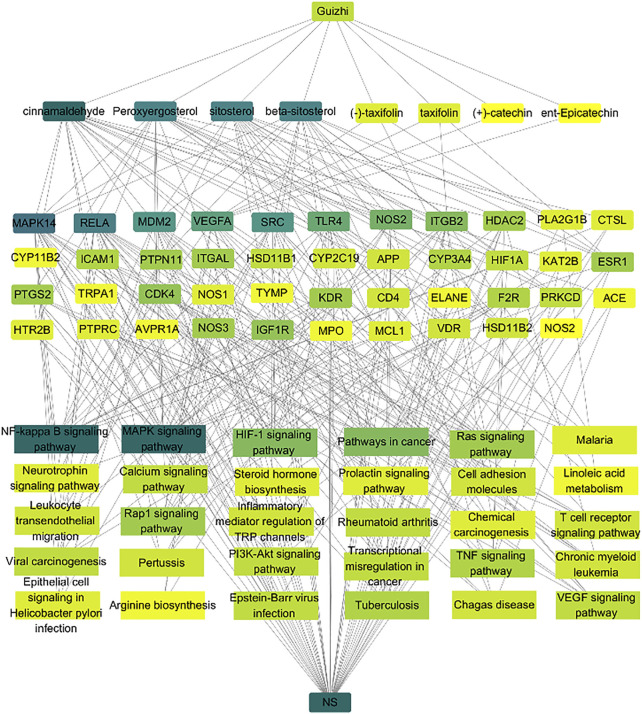
Network analysis of the association among the active compounds, key targets and signaling pathways. Here, we collected the eight active compounds, top 30 KEGG pathways, and build their links with their corresponding targets. The color represented the degree value, and it changes from yellow to green indicated the greater degree value. We found that cinnamaldehyde is the top one active compound with the highest degree value, and the NF-Kappa B and MAPK signaling pathways have more connection with the key targets.

To further investigate the molecular mechanism of CA against NS, an overall network was constructed based on the top 30 significant KEGG signaling pathways and their corresponding targets (Figure 5). 83 nodes (1 drug, eight compound, 43 targets, 30 pathways and 1disease) were contained in this network. The color represented the degree value, and it changes from yellow to green indicated the greater degree value. In the network, CA is the top one active compound with the highest degree value, and in these pathways, NF-Kappa B and MAPK were the most important signaling pathways with the highest degree value. Therefore, the network analysis suggesting that the protective role in kidney of Guizhi might be related to NF-Kappa B and MAPK signaling pathways.

#### Molecular Docking

**FIGURE 6:**
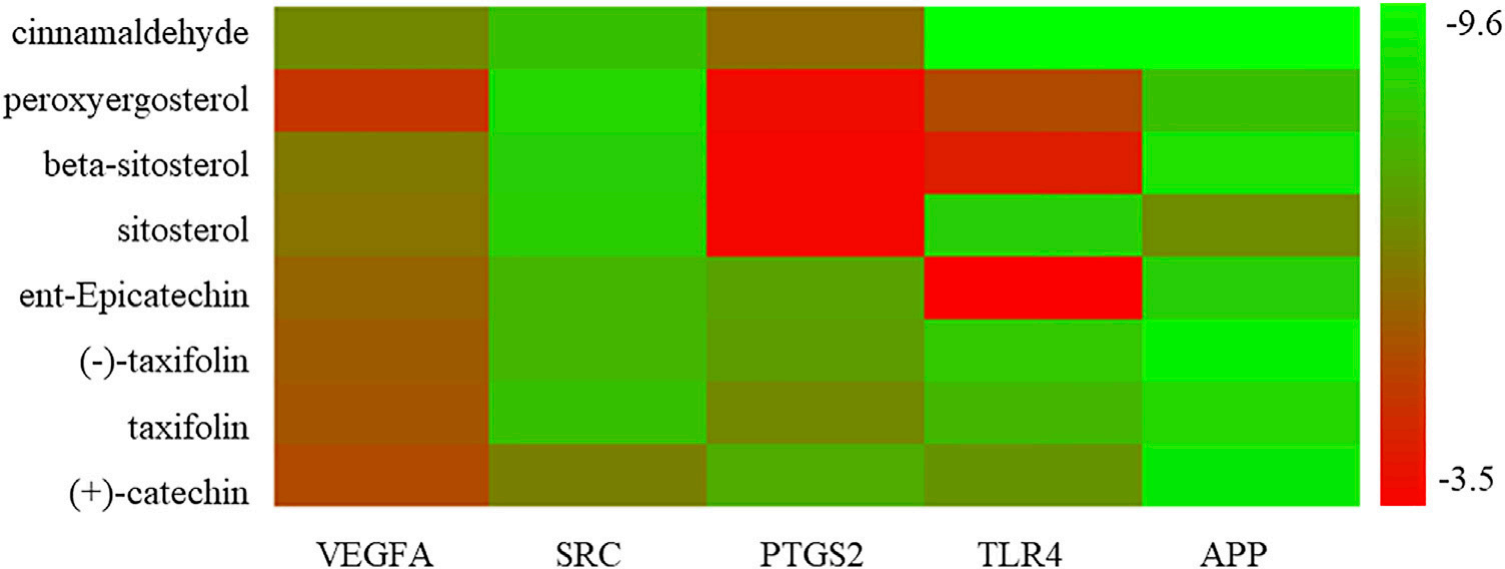
The binding energy of the main active components of Guizhi and the key targets.

**FIGURE 7:**
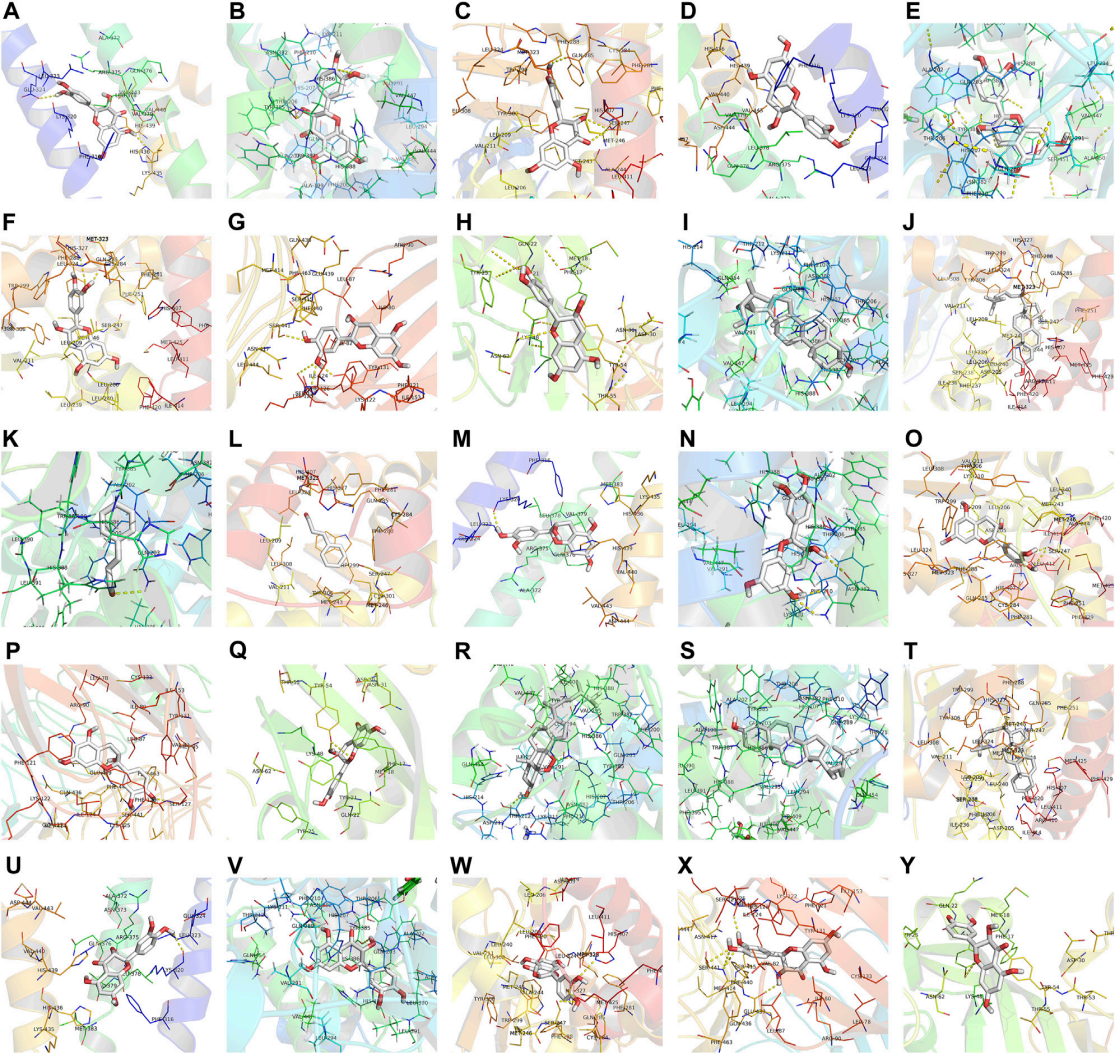
The binding site of the main active components of Guizhi and the key targets. The molecular docking poses of (-)-taxifolin-APP **(A)**, (-)-taxifolin-PTGS2 **(B)**, (-)-taxifolin-SRC **(C)**, (+)-catechin-APP **(D)**, (+)-catechin-PTGS2 **(E)**, (+)-catechin-SRC **(F)**, (+)-catechin-TLR4 **(G)**, (+)-catechin-VEGFA **(H)**, beta-sitosterol-PTGS2 **(I)**, beta-sitosterol-SRC **(J)**, cinnamaldehyde- PTGS2 **(K)**, cinnamaldehyde-SRC **(L)**, ent-Epicatechin-APP **(M)**, ent-Epicatechin-PTGS2 **(N)**, ent-Epicatechin-SRC **(O)**, ent-Epicatechin-TLR4 **(P)**, ent-Epicatechin-VEGFA **(Q)**, peroxyergosterol-PTGS2 **(R)**, sitosterol-PTGS2 **(S)**, sitosterol-SRC **(T)**, taxifolin-APP **(U)**, taxifolin-PTGS2 **(V)**, taxifolin-SRC **(W)**, taxifolin-TLR4 **(X)** and taxifolin-VEGFA **(Y)**.

Molecular docking was performed with the main compounds of Guizhi and the top five important targets in the above PPI network, namely VEGFA, SRC, PTGS2, TLR4 and APP (Table 4). The smaller the affinity value in the docking results, the more stable the interaction between the targets and the active component was. Through molecular docking, it was found that the above five important targets had good binding activities with the active components of Guizhi (Table 5) (Figure 6). Among them, CA showed good binding activity to VEGFA, TLR4 and APP with binding energy of −6.8,−9.6, and−9.6, respectively. Peroxyergosterol showed the best binding activity to SRC with binding energy of −8.7, while (+)-catechin showed good binding activity to PTGS2 for 7.6. The compounds and targets displayed diverse binding patterns at the active sites, including hydrogen bonds, H-π and π-π interactions. And these compounds bind to the targets through interacting with various amino acid residues, such as HIS-436, GLU-324, HIS-386, ALA-199, TYR-385, SER-247, GLN-285. The binding interactions and the binding site of compounds-targets were shown in Figure 7. Interestingly, the docking results between CA and the five key targets revealed that CA has higher binding affinities compared with the other compounds.

**TABLE 4:** The top five targets in PPI network.

NO.	Target name	PDB ID	Degree
1	VEGFA	6BFT	39
2	SRC	6HTY	32
3	PTGS2	5IKV	27
4	TLR4	3FXI	26
5	APP	3UMH	24

**TABLE 5:** Molecular docking results of targets and active components.

Target name	PDB ID	Molecule name	Affinity (kcal/mol)
VEGFA	6BFT	cinnamaldehyde	−6.8
		beta-sitosterol	−6.4
		sitosterol	−6.3
		ent-Epicatechin	−5.9
		(-)-taxifolin	−5.7
		taxifolin	−5.6
		(+)-catechin	−5.4
		peroxyergosterol	−4.9
SRC	6HTY	peroxyergosterol	−8.7
		beta-sitosterol	−8.5
		sitosterol	−8.4
		cinnamaldehyde	−8.1
		taxifolin	−8.1
		(-)-taxifolin	−7.9
		ent-Epicatechin	−7.8
		(+)-catechin	−6.6
PTGS2	5IKV	(+)-catechin	−7.6
		ent-Epicatechin	−7.5
		(-)-taxifolin	−7.3
		taxifolin	−6.8
		cinnamaldehyde	−6.1
		peroxyergosterol	−3.8
		beta-sitosterol	−3.7
		sitosterol	−3.7
TLR4	3FXI	cinnamaldehyde	−9.6
		sitosterol	−8.5
		(-)-taxifolin	−8.3
		taxifolin	−7.9
		(+)-catechin	−7.1
		peroxyergosterol	−5.4
		beta-sitosterol	−4.3
		ent-Epicatechin	−3.5
APP	3UMH	cinnamaldehyde	−9.6
		(-)-taxifolin	−9.3
		(+)-catechin	−9.1
		beta-sitosterol	−9.0
		taxifolin	−8.7
		ent-Epicatechin	−8.5
		Peroxyergosterol	−8.1
		sitosterol	−6.9

### Experimental Verifications *in vitro*

#### MTT Assay

**FIGURE 8:**
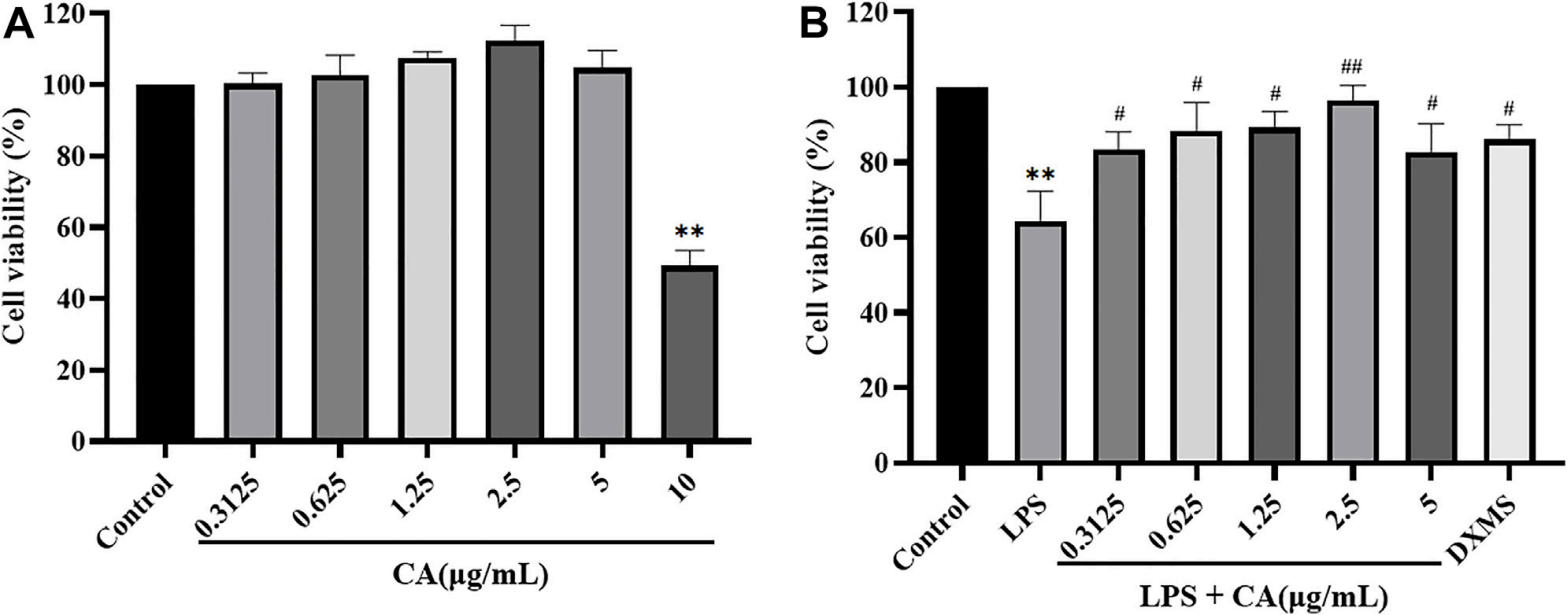
The toxicity efferets after CA treatment in RAW264.7 cells. The effects of CA on RAW264.7 cells cytotoxicity **(A)**; The effects of CA on LPS-induced RAW264.7 cells proliferation ability **(B)**. Cells were pretreated with or without various concentrations of CA for 2 h, and then co-incubated with or without LPS (1 μg/ ml) for 24 h. Cell viability was assessed by MTT. ^**^ *p* < 0.01 *vs*. control group; ^#^ *p* < 0.05, ^##^ *p* < 0.01 *vs*. LPS group.

The results of MTT assays were shown in Figure 8. And it suggested that CA had no inhibitory effect on RAW264.7 cells at the concentration of 5 μg/ml, but had cytotoxicity on RAW264.7 cells at the concentration of 10 μg/ml. Therefore, CA with a concentration of 0.3125–5 μg/ml was chosen to evaluate the proliferation ability of CA on LPS challenged RAW264.7 cells. Even though LPS showed marked cytotoxicity to RAW264.7 cells, treatment with CA significantly enhanced the proliferation capacity in LPS challenged RAW264.7 cells.

#### NO Assay

**FIGURE 9:**
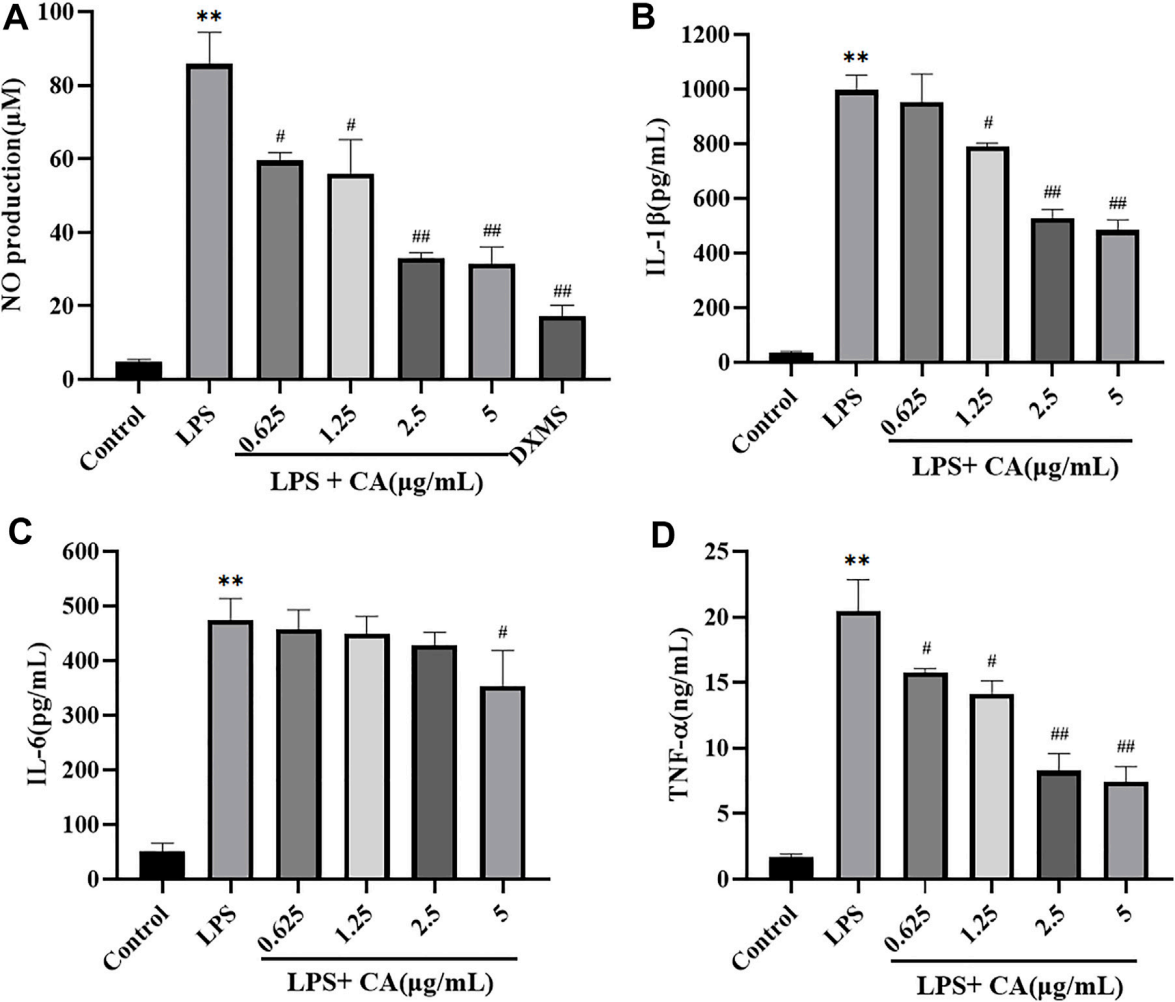
The release of NO **(A)**, IL-1β **(B)**, IL-6 **(C)**, TNF-α **(D)** in RAW264.7 cells. Cells were pretreated with or without various concentrations of CA for 2 h, and then co-incubated with or without LPS (1 μg/ ml) for 24 h. The level of NO in cell culture supernatants was measured using Griess kit. The concentrations of IL-1β, IL-6, and TNF-α in the cell culture medium were detected by ELISA kits. ^**^ *p* < 0.01 *vs*. control group; ^#^ *p* < 0.05, ^##^ *p* < 0.01 *vs*. LPS group.

To investigate the anti-inflammatory of CA, LPS was used to stimulate the release of NO in the RAW264.7 cells to mimic the chronic inflammatory environment. The results (Figure 9A) showed that LPS exposure activated RAW264.7 cells inflammation reflection, as NO secretion in the supernatants significantly enhanced after LPS stimulation for 24 h, and pre-treatment with various concentrations of CA in prior to LPS challenge notably attenuated the enhancement of the cytokine secretions.

#### ELISA of IL-1β, IL-6, TNF-α

The secretion of pro-inflammatory cytokines in LPS challenged RAW264.7 cells were also measured (Figures 9B–D). The levels of IL-1β, IL-6 and TNF-α in cell culture supernatants stimulated with LPS (1 μg/ ml) significantly increased compared to the control group, which indicated that the model of inflammation was successfully established *in vitro*. Administration of CA significantly attenuated the secretion of IL-1β, IL-6 and TNF-α in LPS challenged RAW264.7 cells in a concentration dependent manner. These results indicated that CA exerted anti-inflammatory activity via the suppression of NO production and pro-inflammatory cytokines IL-1β, IL-6 and TNF-α in LPS challenged RAW264.7 cells.

#### Western Blotting

**FIGURE 10:**
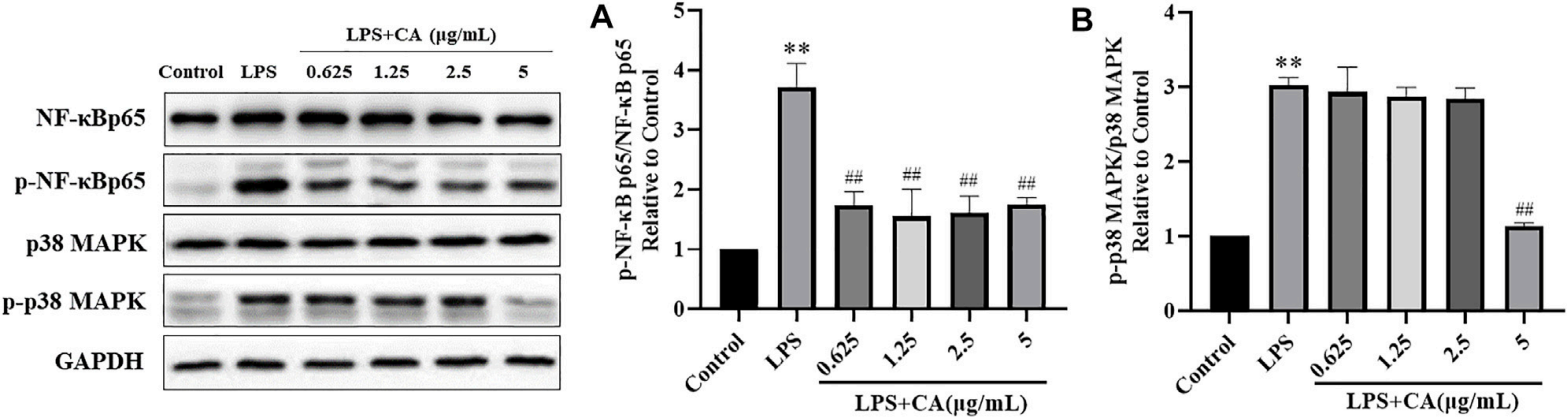
The protein expression of p-NF-κB p65 **(A)** and p-p38 MAPK **(B)**. Cells were pretreated with or without various concentrations of CA for 2 h, and then co-incubated with or without LPS (1 μg/ ml) for 24 h. The levels of protein expression were detected by western blotting. ^*^ *p* < 0.05, ^**^ *p* < 0.01 *vs*. control group; ^##^ *p* < 0.01 *vs*. LPS group.

The major proteins of NF-κB and MAPK signaling pathways were analyzed in LPS challenged RAW264.7 cells. The results showed that NF-κB p65, p-NF-κB p65 and p-p38 MAPK protein levels in LPS stimulated RAW264.7 cells were significantly increased compared to control group. It suggested that NF-κB and MAPK signaling pathways were activated. Treatment with CA significantly decreased the protein expression of NF-κB p65, p-NF-κB p65 and p-p38 MAPK compared to LPS challenged control group (Figure 10). NF-κB p65 and p38 MAPK were mainly localized in the cytosol of RAW264.7 cells, while stimulation with LPS remarkably promoted NF-κB p65 and p38 MAPK translocation into the nucleus. Treatment with CA dramatically lowered the overall translocation of NF-κB p65 and p38 MAPK into the nuclei.

### Experimental Verifications *in vivo*

#### 24 h Urine Volume and Urine Protein

**FIGURE 11:**
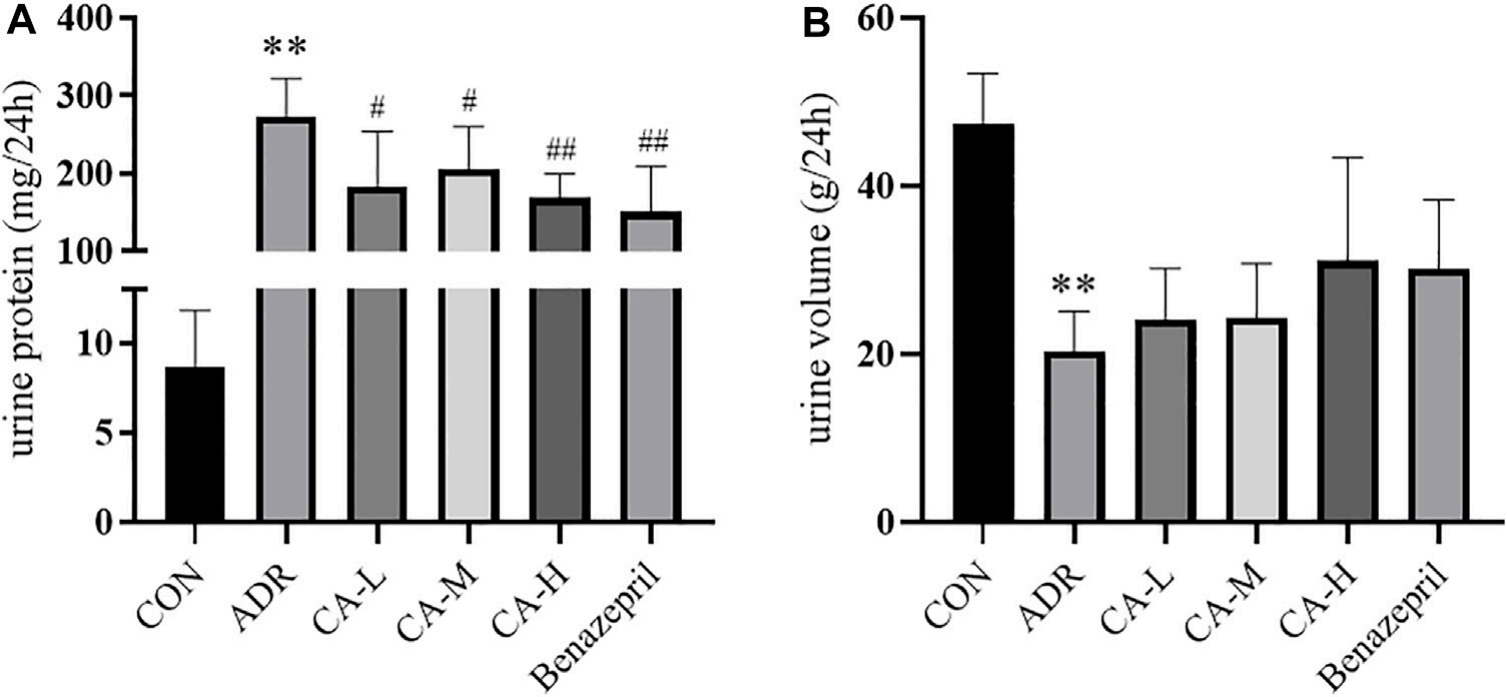
24 h urine volume **(A)** and urine protein content **(B)** in rats. ** *p* < 0.01 *vs* CON group; ^#^ *p* < 0.05, ^##^ *p* < 0.01 *vs* ADR group.

Compared with CON group, 24 h urine volume in ADR group was decreased (*p* <0.01) (Figure 11A), and the urine protein content was increased (*p* <0.01) (Figure 11B). Compared with ADR group, urinary protein in CA-L, CA-M and CA-H groups were decreased (*p* <0.05 or *p* <0.01).

#### Renal Function

**FIGURE 12:**
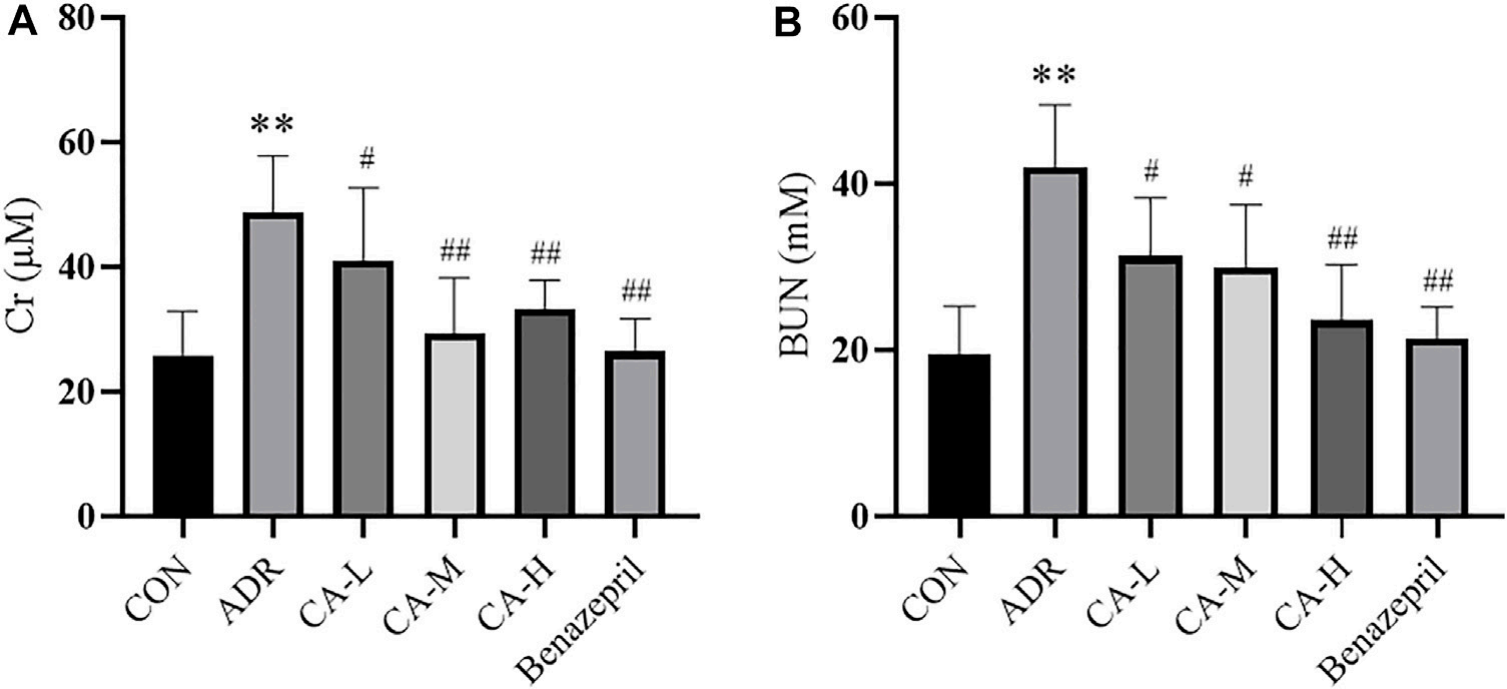
The Cr **(A)** and BUN **(B)** in rats. ** *p* < 0.01 *vs* CON group; ^#^ *p* < 0.05, ^##^ *p* < 0.01 *vs* ADR group.

Compared with CON group, Cr and BUN of ADR group were increased (*p* < 0.01). Compared with ADR group, Cr (Figure 12A) and BUN (Figure 12B) of CA-L, CA-M, and CA-H groups were decreased (*p*<0.05 or *p*<0.01).

#### Western Blot

**FIGURE 13:**
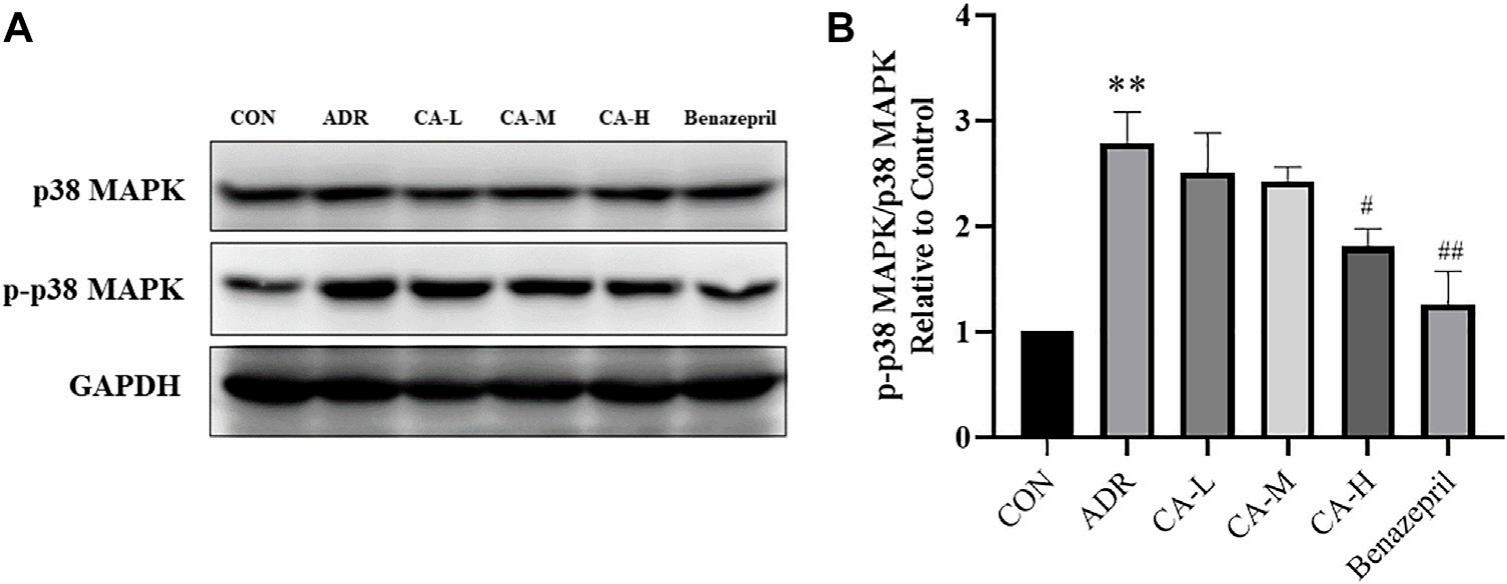
The protein expression of p-p38 MAPK **(A)** was detected by western blotting, and the gray value was calculated by Image J **(B)**. ** *p* < 0.01 *vs*. CON group; ^#^ *p* < 0.05, ^##^ *p* < 0.01 vs. ADR group.

In western blot analysis, compared with the CON group, p-p38 MAPK in ADR group were increased. Compared with ADR group, p-p38 MAPK were decreased in CA-L, CA-M and CA-H groups (Figures 13A,B).

## Discussion

Epidemiological survey reveals that the incidence of NS is about 2–10/100,000, and mostly occurred in male children (Sinha and Bagga, 2012; Lombel et al., 2013). According to the study, the pathogenesis of NS mainly related to the inflammatory response (Singbartl et al., 2019). With multiple complications, poor efficacy and high recurrence rate (He, 2016), NS brings great pressure to people’s life and health. Therefore, exploring the pathogenesis of NS has become increasing urgency.

Guizhi is a drug which has been included in traditional Chinese medicine (TCM) for more than 2000 years with the actions of warming Yang to promote blood circulation and water metabolism. It has been verified in modern pharmacological research that Guizhi is of actions of anti-inflammation, diuresis, invigorate blood circulation and dilate blood vessels, etc. Guizhi is the best known drug in TCM to formulate many formulas which are still commonly used nowadays, such as Wuling San, which is frequently used in clinic to treat chronic nephritic diseases. Guizhi is believed to play a critical role in Wuling San, and our serial researches are stressed on Guizhi and the active ingredient cinnamaldehyde (CA). CA is the main active component of Guizhi with pharmacological activities of anti-inflammation, antihypertension, vascular endothelial protection, etc.

Our previous study suggested that CA was the main active component of Guizhi, and played a key role in Wuling San against kidney injury in rats nephropathy stimulated by adriamycin (Zhang et al., 2019).

However, it is not clear that whether there are other unknown active components in Guizhi that also play a crucial role in the treatment of nephrotic syndrome and what is underlying mechanism of the unknown active components against nephrotic syndrome?

Therefore, the main active components of Guizhi and the possible action mechanism to treat nephrotic syndrome were predicted by network pharmacology. It was found that CA indeed had the highest degree value in the overall network, and the NF-Kappa B and MAPK signaling pathways ranked the first In KEGG enrichment analysis and the overall network. Therefore, CA was reasonably selected as the most effective ingredient of Guizhi in the *in vitro* and *in vivo* research.

To validate the results of network pharmacology, NF-Kappa B, MAPK signaling pathway and many inflammatory cytokines, such as NO, IL-1β, IL-6, and TNF-α, were selected for the *in vitro* verification. Subsequently, LPS induced inflammation in RAW264.7 cells was applied to simulate inflammation in order to investigate the underlying action mechanisms of Guizhi to treat NS.

Lipopolysaccharide (LPS) is the main component of the outer membrane of Gram-negative bacteria, which is one of the factors that cause systemic inflammation response (Lu et al., 2008; Takeuchi and Akira, 2010). Macrophages are important cells that involved in inflammatory response and play a critical role in the pathogenesis of inflammatory diseases (Van den Bossche et al., 2017). In many pathologic paradigms of kidney injuries, macrophages are the major inflammatory effectors (Duffield, 2010). And a strong association between macrophage infiltration and renal disease prognosis has been demonstrated in several human biopsy studies (Eardley et al., 2008). In many studies, LPS stimulated RAW264.7 cells were used to simulate inflammation associated with kidney disease *in vitro* (Chen et al., 2015).

LPS treatment *in vitro* results in an inflammatory response and increased secretion of many pro-inflammatory factors such as TNF-α and NO in mouse macrophage RAW264.7 cells (Lee et al., 2009). NO is an inflammatory molecule that plays a dual role in acute kidney injury (Chen X. et al., 2019). Excess NO can react with superoxide radical to form peroxynitrite, which can cause renal toxicity. NF-κB is also a pivotal factor in controlling the expression of many inflammatory genes (Liu et al., 2017). When exposed to inflammatory stimulant, free NF-κB translocates from the cytoplasm to the nucleus, and phosphorylated subunit p65 stimulates transcription of pro-inflammatory cytokine IL-1β, IL-6, and TNF-α, which promote their secretion and triggering an increased inflammatory response (Peerapornratana et al., 2019), and thus play a key role in the pathogenesis of kidney disease (Liu et al., 2018). However, these pro-inflammatory cytokines can activate the NF-κB signaling pathway in turn, which can increase the expression of many other inflammatory cytokines and subsequently exacerbate the inflammatory response in kidney disease (Dellepiane et al., 2016; Gómez and Kellum, 2016).

In the current study, it was demonstrated that LPS exposure activated RAW264.7 cells inflammation reflection, as NO, IL-1β, IL-6, and TNF-α secretion in the supernatants significantly enhanced after LPS stimulation for 24 h. In contrast, treatment with CA significantly inhibited the levels of NO, IL-1β, IL-6, and TNF-α in RAW264.7 cells that challenged by LPS. These results indicated that CA exerts anti-inflammatory activity via the suppression of NO, IL-1β, IL-6, and TNF-α secretion in LPS challenged RAW264.7 cells.

NF-κB, a transcription factor that responds to and regulates inflammation and immunity, can quickly respond to various inflammatory stimuli, and has attracted extensive attention for its role in regulating renal damage and the progression of renal disease (Chade et al., 2020). Studies have shown that inflammation plays a key role in the pathogenesis of NS and closely relates to the activation of NF-κB signaling pathway (Zhai et al., 2019). NF-κBp65 is activated in the development and progression of renal disease (Zhang P. et al., 2021), and inhibition of NF-κB signaling pathway can reduce the proteinuria and kidney injury (Lu et al., 2019; Zhai et al., 2019). NF-κB activation is widely implicated in inflammatory diseases and there has been substantial attention on the development of anti-inflammatory drugs targeting NF-κB (Karin et al., 2004).

Renal disease is closely related to inflammation, and NF-κB, as well as its upstream regulator mitogen-activated protein kinases (MAPKs) are activated when inflammatory response is activated (Ren et al., 2020). The activation of MAPK signaling pathways can induce the apoptosis of renal proximal tubule epithelial cells, promote inflammation and renal injury (Chen Y. et al., 2019). And the activation of p38MAPK, a member of the family of MAPKs, plays a key role in kidney inflammation by promoting production of pro-inflammatory cytokines and regulating apoptosis during renal injury (Chang et al., 2018).

Our results showed that, the phosphorylation levels of NF-κBp65 and p38 MAPK protein level were increased in the LPS-treated control group. However, the phosphorylation levels of NF-κBp65 and p38 MAPK in CA pretreated RAW264.7 cells were decreased to a certain extent, indicating that the effect of Guizhi against NS might mainly achieved through NF-Kappa B and MAPK signaling pathways.

ADR-induced renal injury in rats is the classical model of NS (Bertani et al., 1982). *In vivo* experiment indicated that, CA can reduce the urinary protein, Cr and BUN in rats induced by ADR. In western blot, the phosphorylation levels of p-p38 MAPK in CA treated rats were decreased. However, it is found that the phosphorylation levels of NF-κBp65 was underexpressed.

Taken together, we found that CA might be the most important active compound of Guizhi, the action mechanisms of Guizhi against NS was related to inflammation, and might mainly through MAPK signaling pathway. However, the present study also has some limitations. First, the public online databases we used in this research are imperfect and constantly updated yet, some of the active ingredients, targets and signaling pathways might not be included in the analysis. Moreover, the other signaling pathways, such as VEGF, PI3K-Akt, RAS signaling pathways, might also play important roles in Guizhi against NS. Therefore, further studies are expected to explore the more underlying mechanism.

## Conclusion

In summary, the underlying action mechanisms of Guizhi against NS was explored via the integration of network pharmacology and *in vitro*, *in vivo* experimental verification. The results showed that CA might be the main active component of Guizhi against NS, and the underlying mechanism might mainly be achieved by inhibiting MAPK signaling pathways, and thereby inhibiting the release of NO, IL-1β, IL-6 and TNF-α in LPS challenged RAW264.7 cells, suggesting that CA might be a potential compound for the development of novel and promising approach against NS.

## Data Availability

The original contributions presented in the study are included in the article/Supplementary Material, further inquiries can be directed to the corresponding author.
